# Supplementary material for: Nuclear Magnetic Resonance Metabolomics Approach for the Analysis of Major Legume Sprouts Coupled to Chemometrics
Source: Molecules. 2021 Feb 2;26(3):761. doi: 10.3390/molecules26030761 (PMC7867271; doi:10.3390/molecules26030761)
Supplement: Supplementary file 1 [file molecules-26-00761-s001.pdf]

Comparative Nuclear Magnetic Resonance Metabolomics Approach for the Analysis of Major Legume Sprouts Coupled to Chemometrics

Mohamed A. Farag[1,2*], Mohamed G. Sharaf El-Din[3], Mohamed A. Selim[1,4], Asmaa I. Owis[5], Sameh F. Abouzid[5], Andrea Porzel[6], Ludger A. Wessjohann[6*], Asmaa Otify[1]

[1] Pharmacognosy Department, Faculty of Pharmacy, Cairo University, Cairo, Egypt; mfarag73@yahoo.com (M.A.F); dr_nabilselim79@yahoo.com (M.A.S.); asmaa.otify@gmail.com (A.O)

[2] Chemistry Department, School of Sciences & Engineering, The American University in Cairo, New Cairo, Egypt; mohamed.alifarag@aucegypt.edu

[3] Pharmacognosy Department, Faculty of Pharmacy, Port Said University, Port Said, Egypt; drmgm4824@yahoo.com

[4] Pharmacognosy Department, Faculty of Pharmacy, Misr University for Science & Technology (MUST), 6th October City, Egypt; dr_nabilselim79@yahoo.com

[5] Pharmacognosy Department, Faculty of Pharmacy, Beni-Suef University, Beni-Suef, Egypt; asmaa_owis@yahoo.com (A.I.O.); sameh.zaid@pharm.bsu.edu.eg (S.F.A)

[6] Department of Bioorganic Chemistry, Leibniz Institute of Plant Biochemistry, Weinberg 3, 06120 Halle (Saale), Germany; aporzel@ipb-halle.de (A.P.); wessjohann@ipb-halle.de (L.A.W.)

[*] Corresponding authors: Prof. Dr. Mohamed Farag mfarag73@yahoo.com, Prof. Dr. Ludger Wessjohann wessjohann@ipb-halle.de

Table S1: ^{1}H-NMR signals used in major metabolites identified by quantitative NMR analysis.

ID	Compound	Protons used in quantification
**1**	**ω-6 Fatty acid**	t-CH_3
**2**	**ω-3 Fatty acid**	t-CH_3
**3**	**Sucrose**	H-1
**4**	**Fructose**	H-3
**5**	***α*-Glucose**	H-1
**6**	***β*-Glucose**	H-1
**7**	**Alanine**	H-3
**8**	**Valine**	H-5
**10**	**4-Hydroxyisoleucine**	H-5
**11**	**Asparagine**	H-2a
**12**	**Choline**	*N*-$(CH_3)_3$
**13**	**Betaine**	*N*-$(CH_3)_3$
**15**	**Phenylalanine**	H-2′/H-6′
**16**	**Tyrosine**	H-2′/H-6′
**17**	**L-dopa**	H-3a
**18**	**Tryptophan**	H-5
**19**	**Histidine**	H-6
**20**	**Cytosine**	H-6
**22**	**Acetic acid**	CH_3
**23**	**Fumaric acid**	H-2/H-3
**24**	***β*-Sitosterol**	H-18
**25**	**Trigonelline**	H-2
**26**	**Biochanin A**	H-6
**27**	**Genistin**	H-6
**28**	**Malonyl-genistin**	H-6
**29**	**Formononetin**	H-6
**30**	**Daidzin**	H-8
**31**	**Malonyl-daidzin**	H-8
**32**	**Cicerin**	H-8

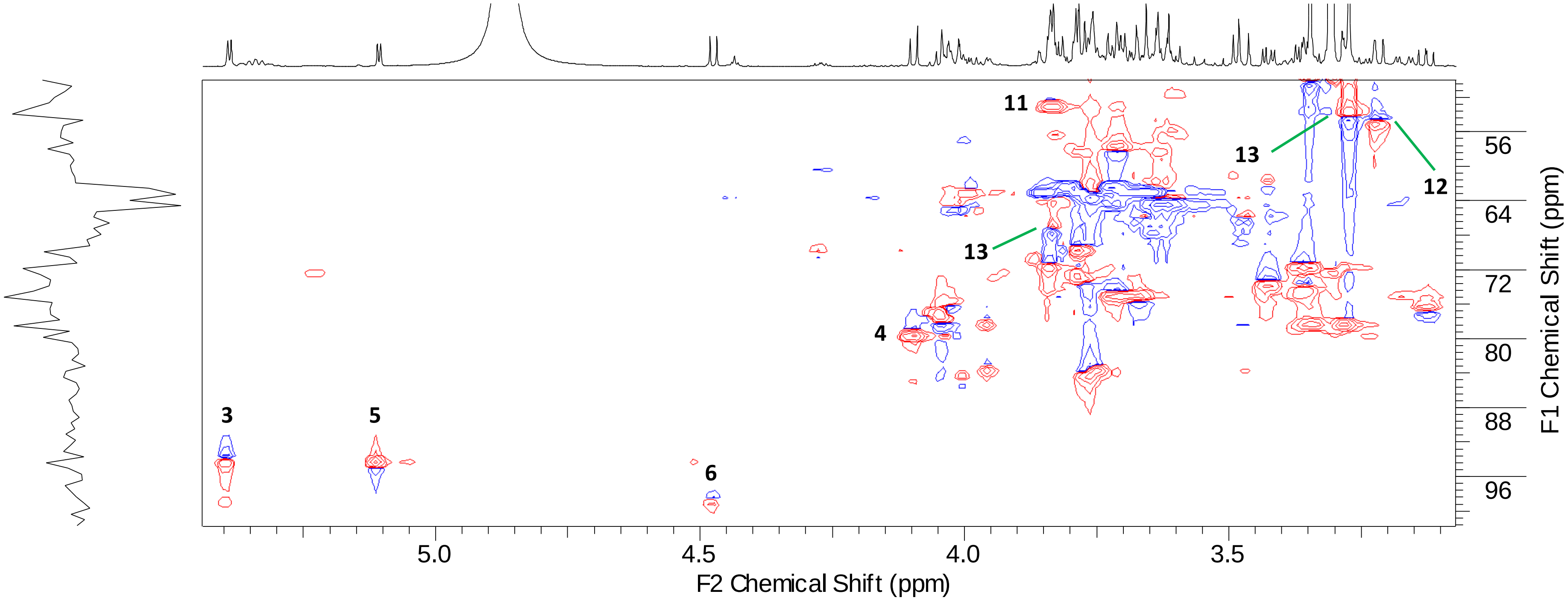

Figure S1. Signal assignment of the ^{1}H-NMR markers for compounds **3-6** & **11-13** signals using ^{1}H-^{13}C correlations observed in the HSQC spectrum of *Vicia* sprouts methanol extract. Peaks assigned in the spectrum are labeled as follows: Sucrose (**3**), fructose (**4**), *α*-glucose (**5**), *β*-glucose (**6**), asparagine (**11**), choline (**12**), and betaine (**13**).

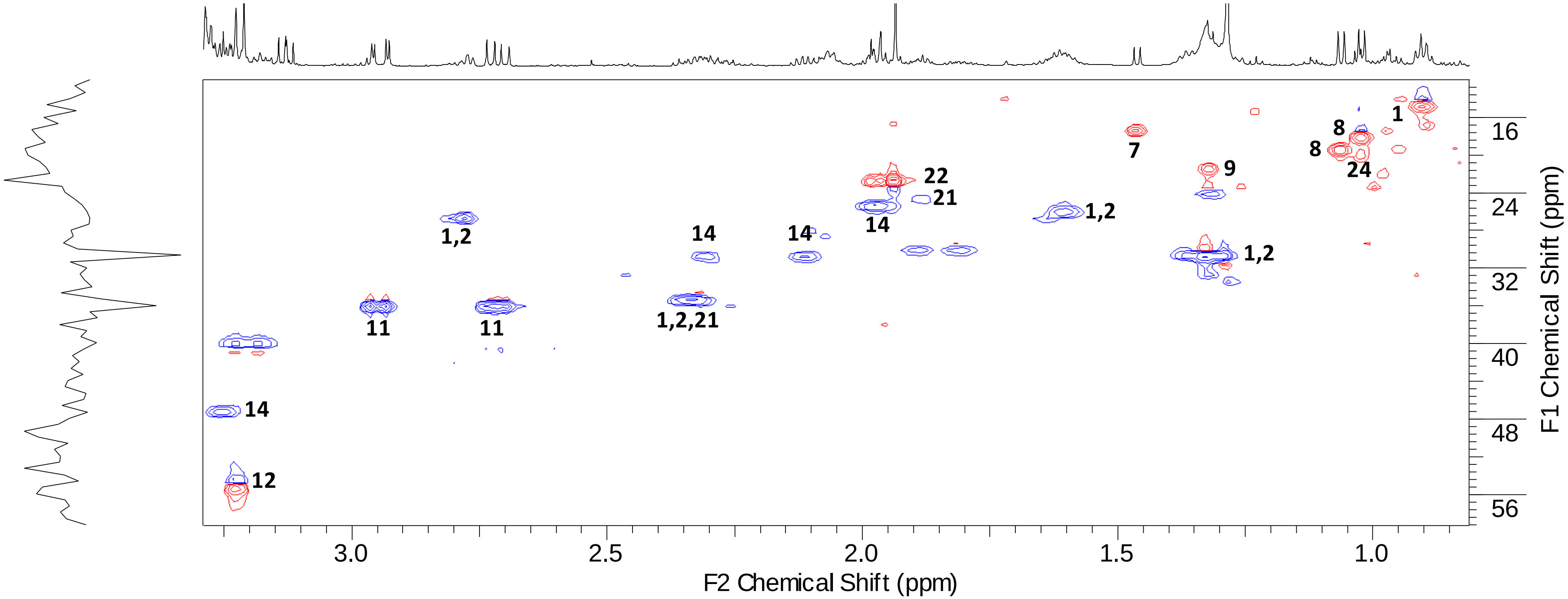

Figure S2. Signal assignment of the ^{1}H-NMR markers for compounds **1**, **2**, **7-9**, **11-12**, **14**, **21-22** & **24** signals using ^{1}H-^{13}C correlations observed in the HSQC spectrum of *Lens* sprouts methanol extract. Peaks assigned in the spectrum are labeled as follows: ω-6 fatty acid (**1**), ω-3 fatty acid (**2**), alanine (**7**), valine (**8**), threonine (**9**), asparagine (**11**), choline (**12**), proline (**14**), 4-aminobutyric acid (**21**), acetic acid (**22**), and *β*-sitosterol (**24**).

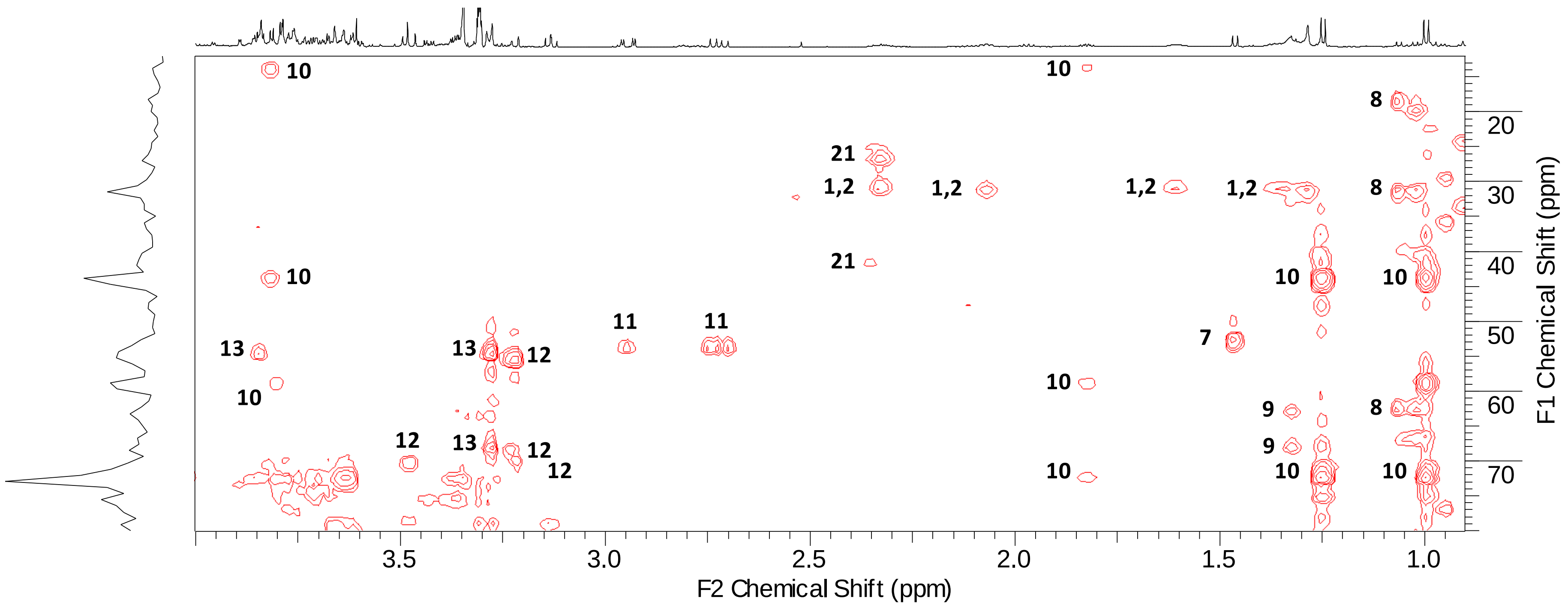

Figure S3. Signal assignment of the ^{1}H-NMR markers for compounds **1-2**, **7-13** & **21** signals using ^{1}H-^{13}C correlations observed in the HMBC spectrum of *Trigonella* sprouts methanol extract. Peaks assigned in the spectrum are labeled as follows: ω-6 fatty acid (**1**), ω-3 fatty acid (**2**), alanine (**7**), valine (**8**), threonine (**9**), 4-hydroxy-isoleucine (**10**), asparagine (**11**), choline (**12**), betaine (**13**), and 4-aminobutyric acid (**21**).

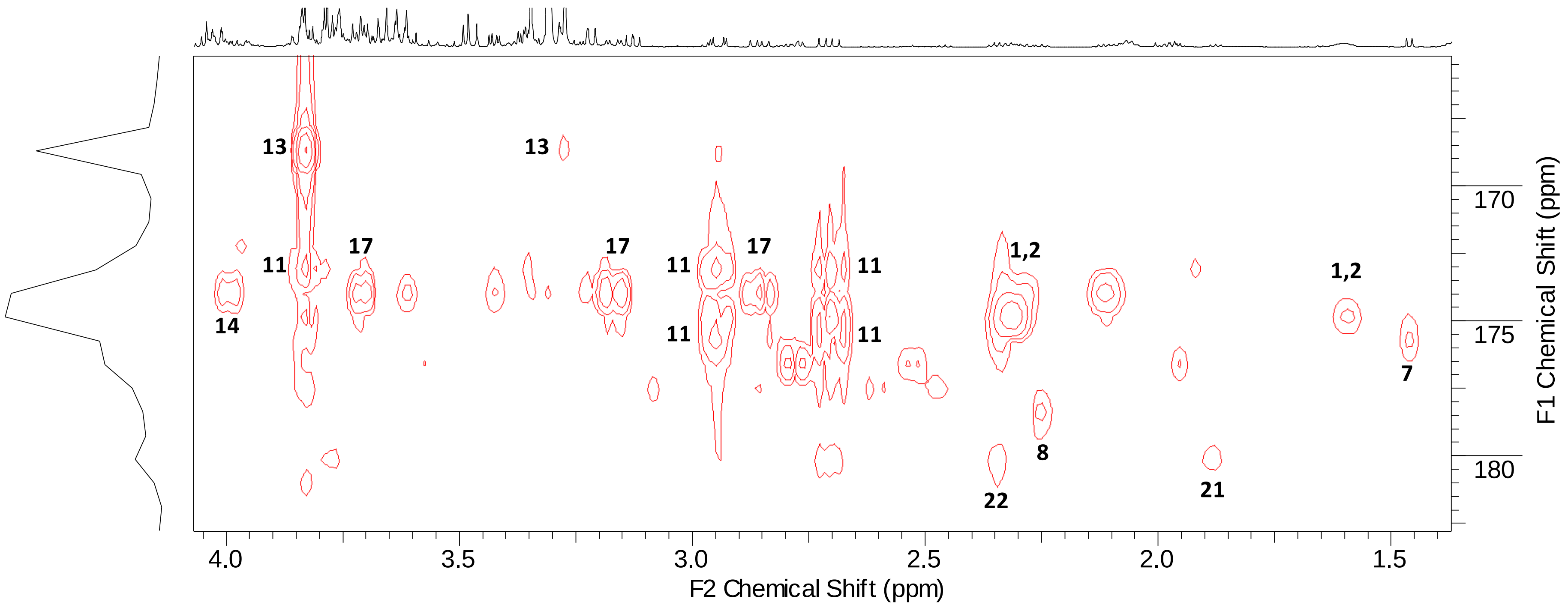

Figure S4. Signal assignment of the ^{1}H-NMR markers for compounds **1-2**, **7-8**, **11**, **13-14**, **17** & **21** signals using ^{1}H-^{13}C correlations observed in the HMBC spectrum of *Vicia* sprouts methanol extract. Peaks assigned in the spectrum are labeled as follows: ω-6 fatty acid (**1**), ω-3 fatty acid (**2**), alanine (**7**), valine (**8**), asparagine (**11**), betaine (**13**), proline (**14**), L-dopa (**17**), and 4-aminobutyric acid (**21**).

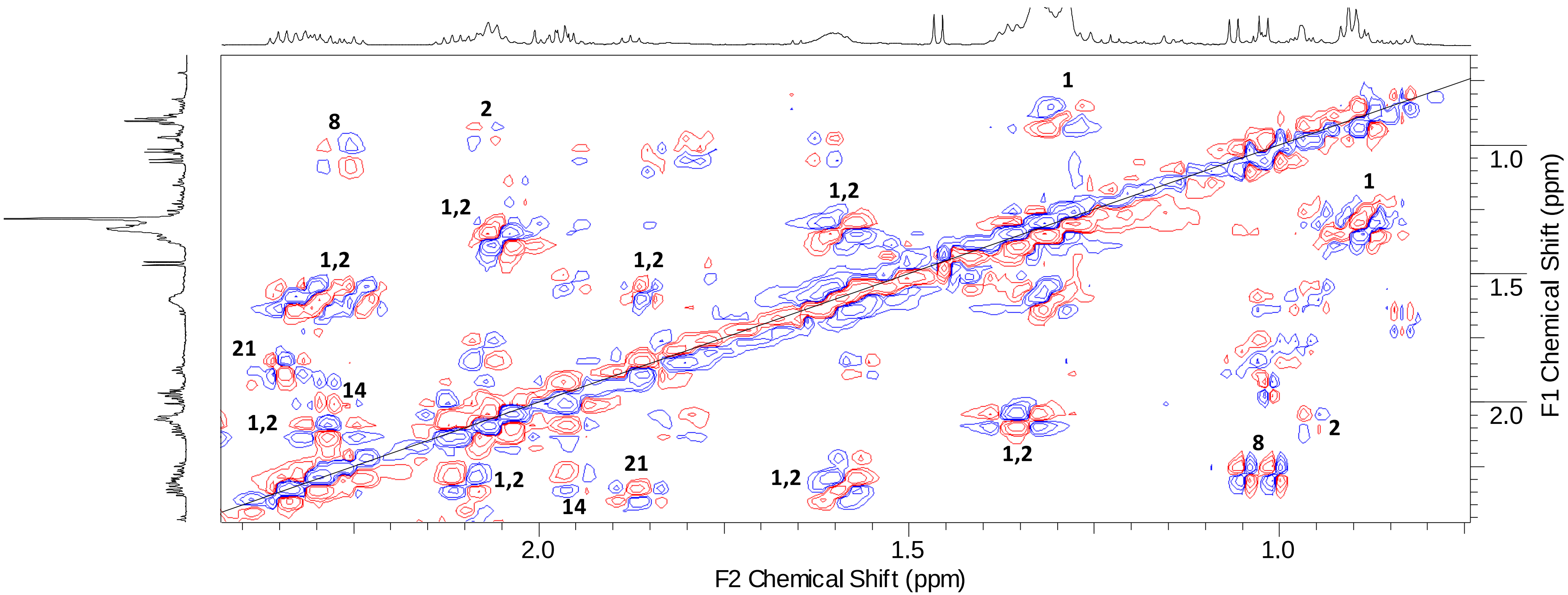

Figure S5. ^{1}H-^{1}H COSY spectrum of *Vicia* sprouts methanol extract showing correlations observed for compounds **1-2**, **8**, **14**, & **22** that were used to confirm the assignments of their signals observed in ^{1}H-NMR spectrum. Peaks assigned in the spectrum are labeled as follows: ω-6 fatty acid (**1**), ω-3 fatty acid (**2**), valine (**8**), proline (**14**), and 4-aminobutyric acid (**21**).

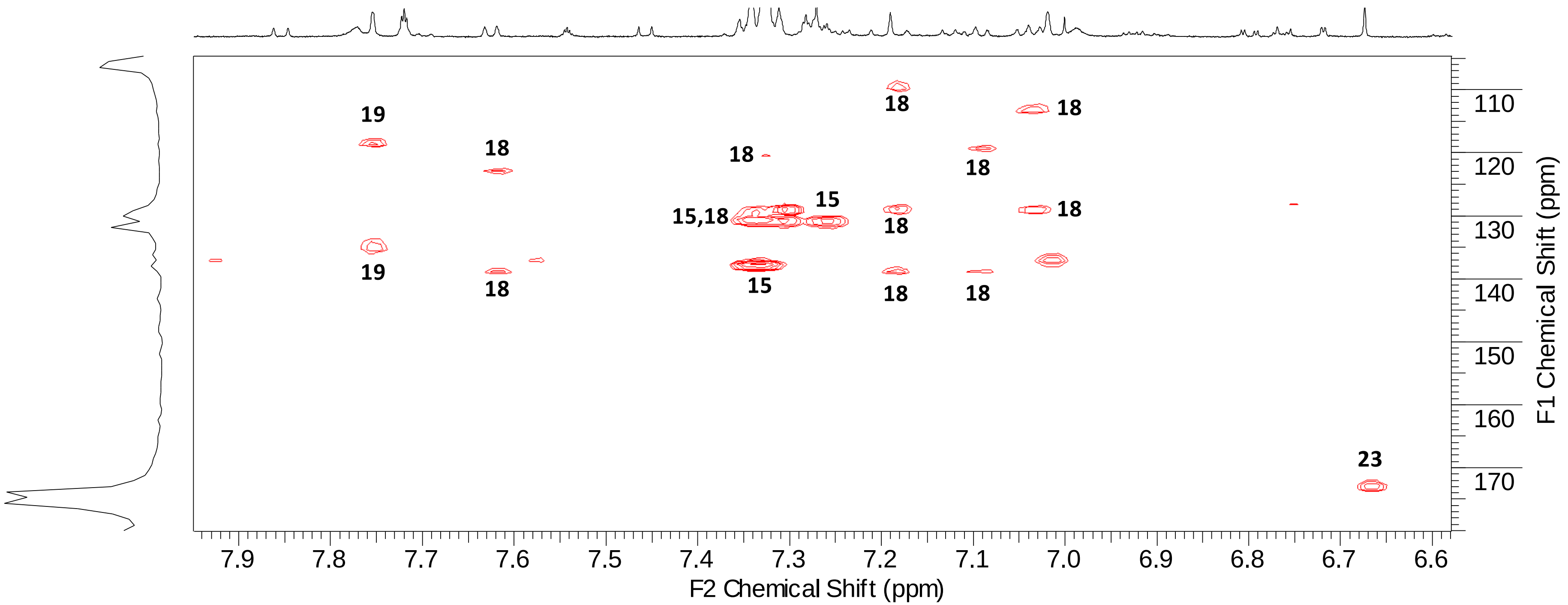

Figure S6. Signal assignment of the ^{1}H-NMR markers for compounds **15**, **18**-**19** & **23** signals using ^{1}H-^{13}C correlations observed in the HMBC spectrum of *Lens* sprouts methanol extract. Peaks assigned in the spectrum are labeled as follows: Phenylalanine (**15**), tryptophan (**18**), histidine (**19**), and fumaric acid (**23**).

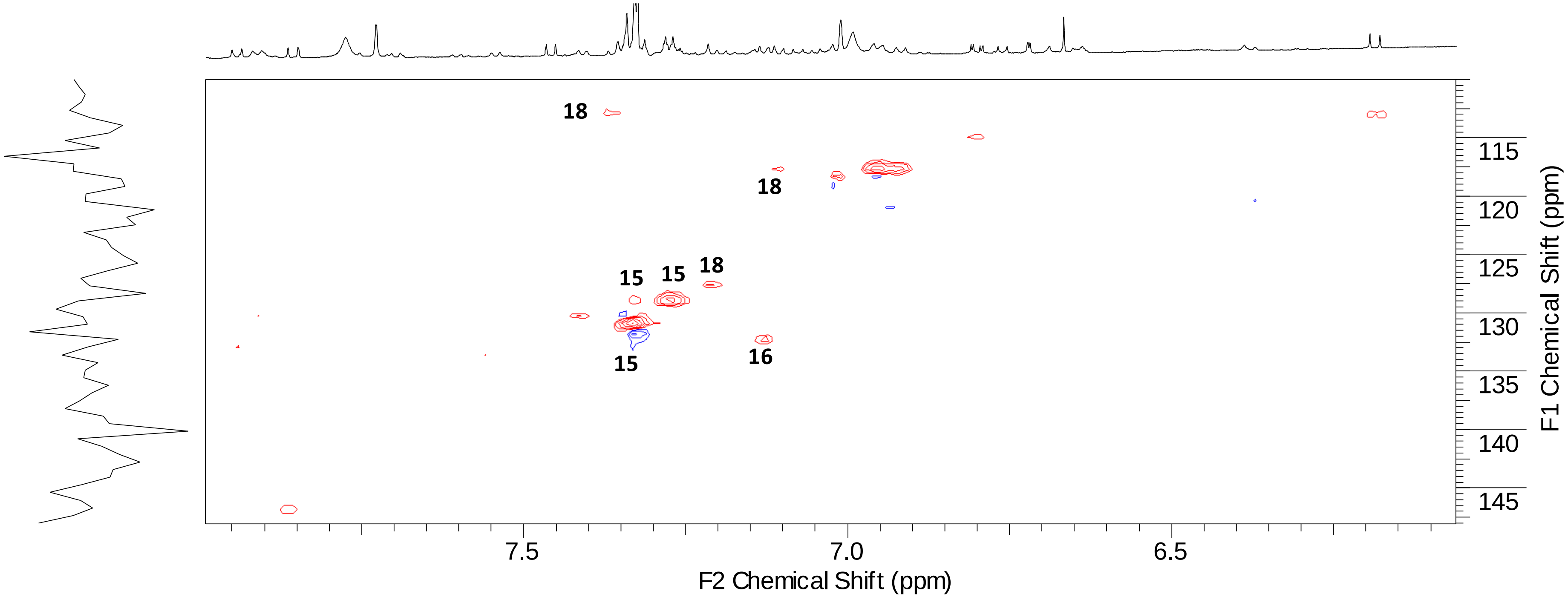

Figure S7. Signal assignment of the ^{1}H-NMR markers for compounds **15**, **16** & **18** & signals using ^{1}H-^{13}C correlations observed in the HSQC spectrum of *Trigonella* sprouts methanol extract. Peaks assigned in the spectrum are labeled as follows: Phenylalanine (**15**), tyrosine (**16**), and tryptophan (**18**).

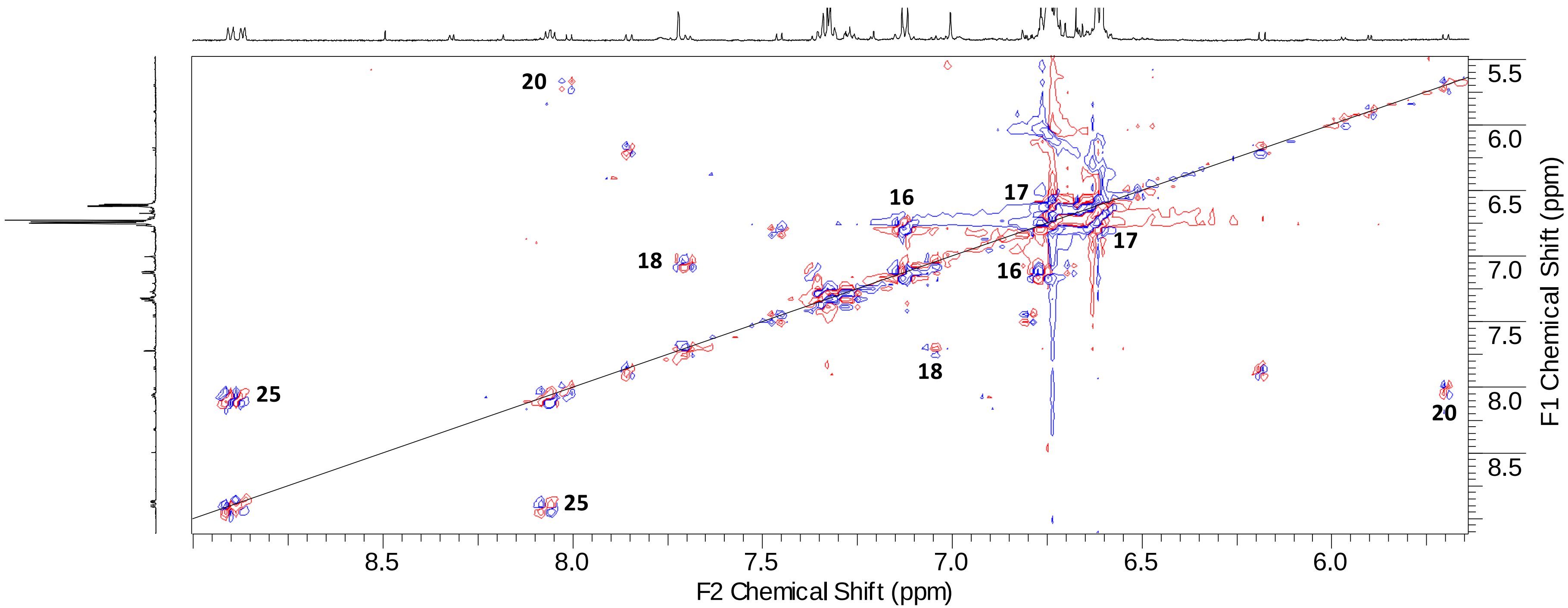

Figure S8. ^1H-^1H COSY spectrum of *Vicia* sprouts methanol extract showing correlations observed for compounds **16-18**, **20**, & **25-26** that were used to confirm the assignments of their signals observed in ^1H-NMR spectrum. Peaks assigned in the spectrum are labeled as follows: Tyrosine (**16**), L-dopa (**17**), tryptophan (**18**), cytosine (**20**), and trigonelline (**25**).

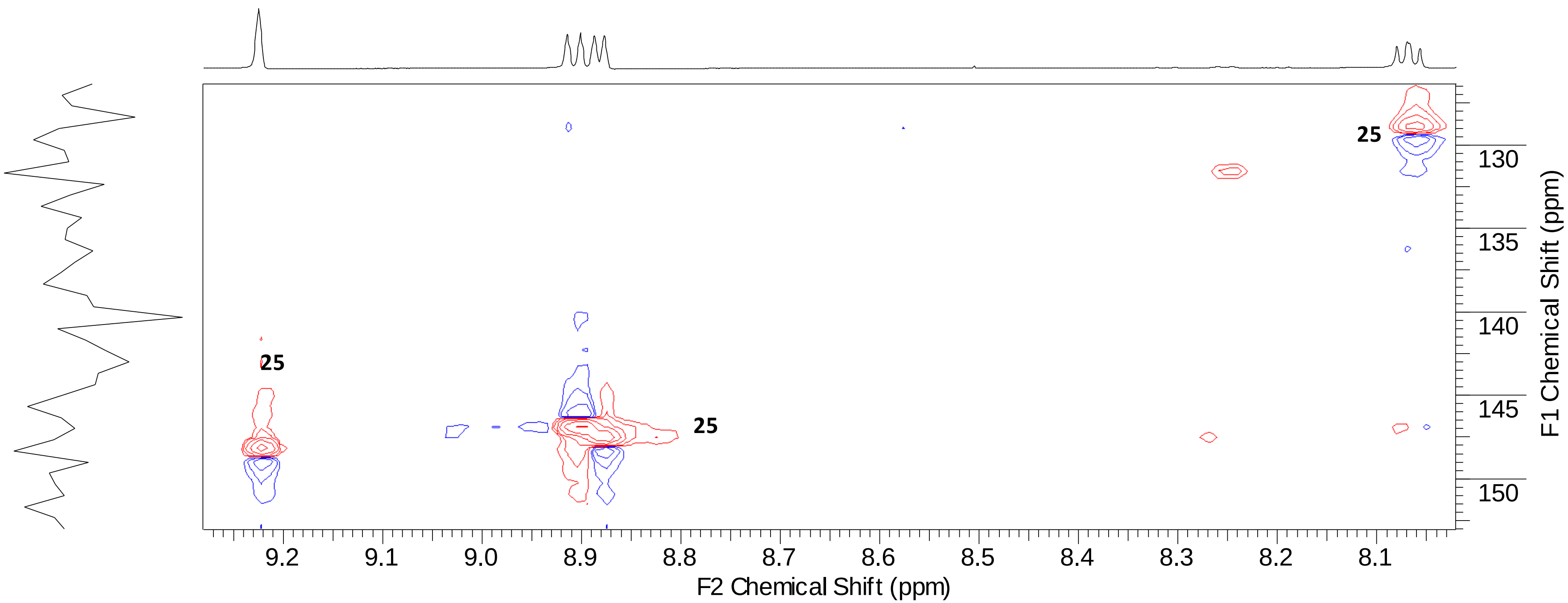

Figure S9. Signal assignment of the ^{1}H-NMR markers for trigonelline (**25**) signals using ^{1}H-^{13}C correlations observed in the HSQC spectrum of *Trigonella* sprouts methanol extract.

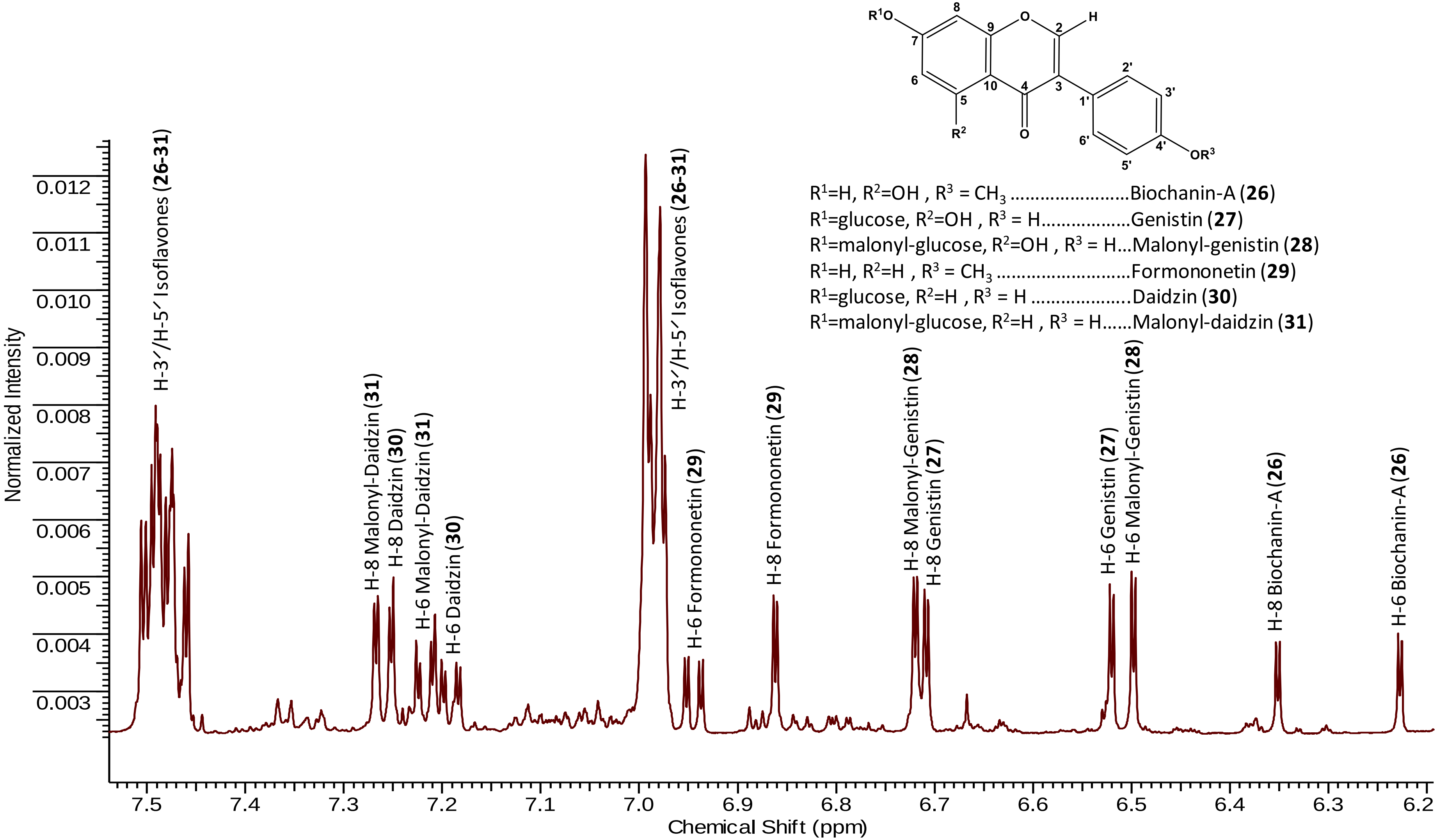

Figure S10. Signal assignment of the ¹H-NMR markers for isoflavones **26-31** observed in the ¹H-NMR spectrum of *Cicer* sprouts methanol extract.

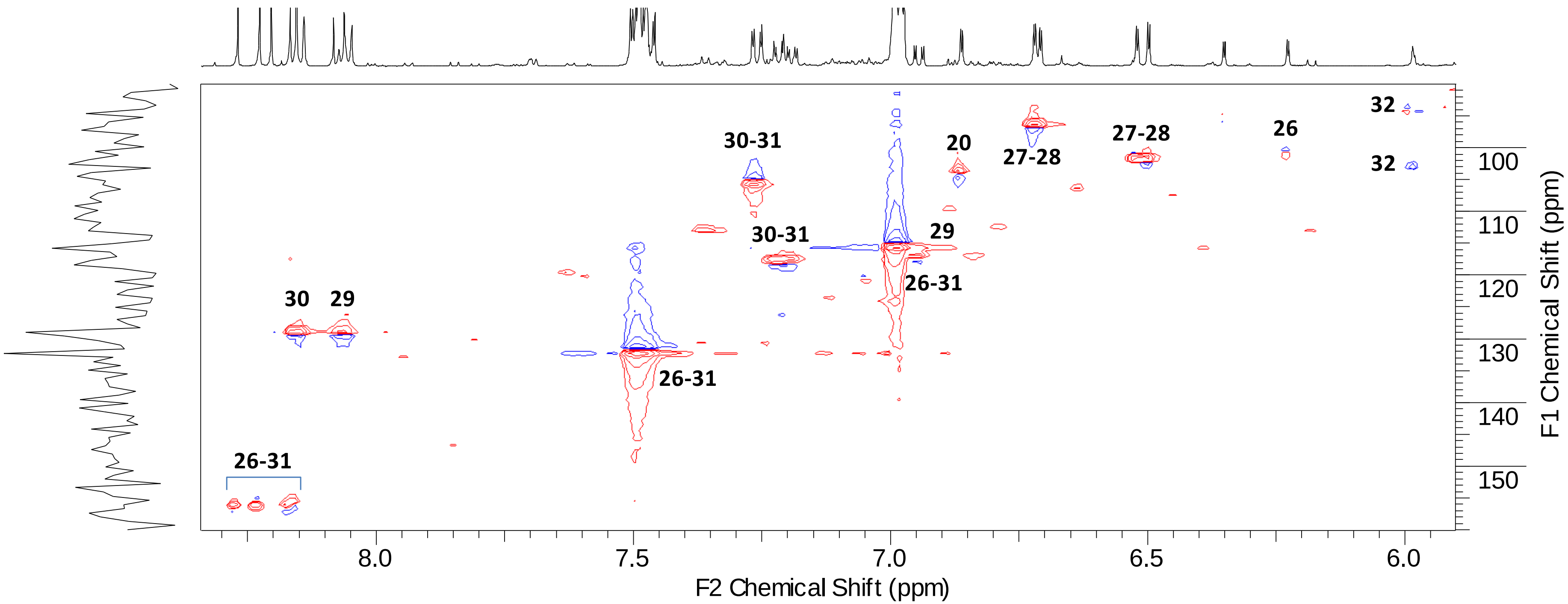

Figure S11. Signal assignment of the ^{1}H-NMR markers for isoflavonoids **26**-**32** signals using ^{1}H-^{13}C correlations observed in the HSQC spectrum of *Cicer* sprouts methanol extract. Peaks assigned in the spectrum are labeled as follows: Biochanin-A (**26**), genstin (**27**), malonyl-genstin (**28**), formononetin (**29**), daidzin (**30**), malonyl-daidzin (**31**), and cicerin (**32**).

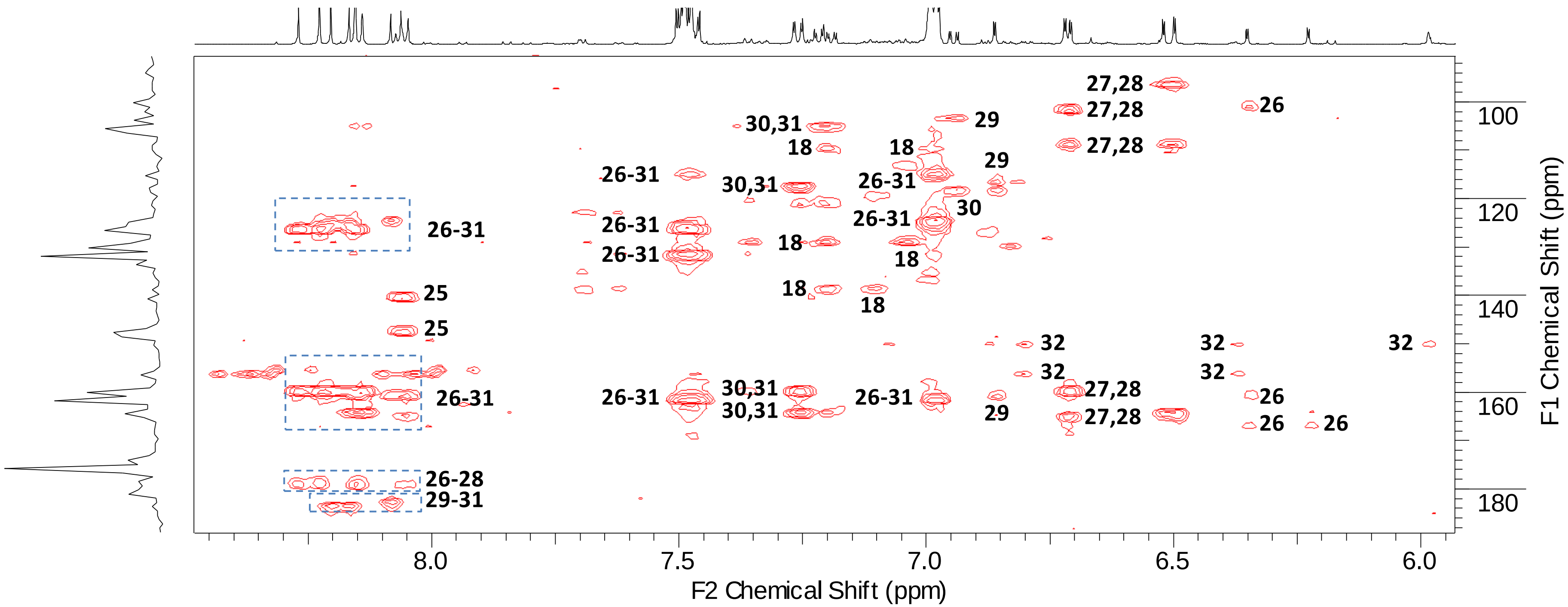

Figure S12. Signal assignment of the ^{1}H-NMR markers for compounds **18** & **25**-**32** signals using ^{1}H-^{13}C correlations observed in the HMBC spectrum of *Cicer* sprouts methanol extract. Peaks assigned in the spectrum are labeled as follows: Tryptophan (**18**), trigonelline (**25**), biochanin-A (**26**), genistin (**27**), malonyl-genstin (**28**), formononetin (**29**), daidzin (**30**), malonyl-daidzin (**31**), and cicerin (**32**).

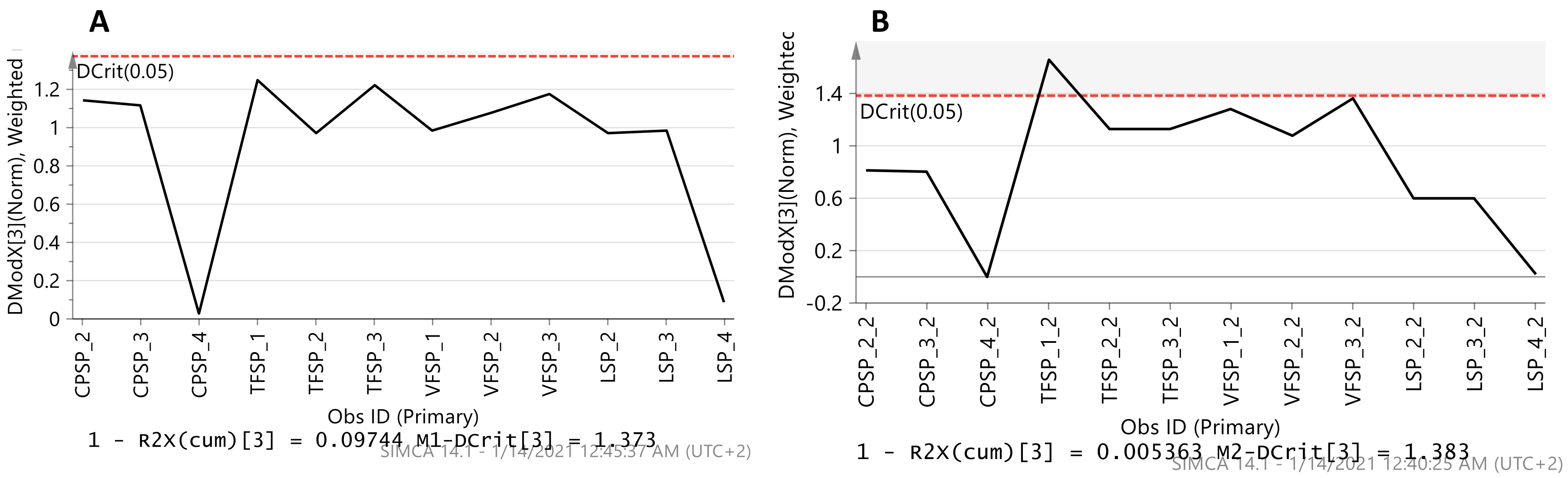

Figure S13. The DModX plot of the PCA model in **A.** full (δ 11.0-0.0 ppm) and **B.** aromatic (δ 11.0-5.0 ppm) regions showing the moderate outliers (in red).

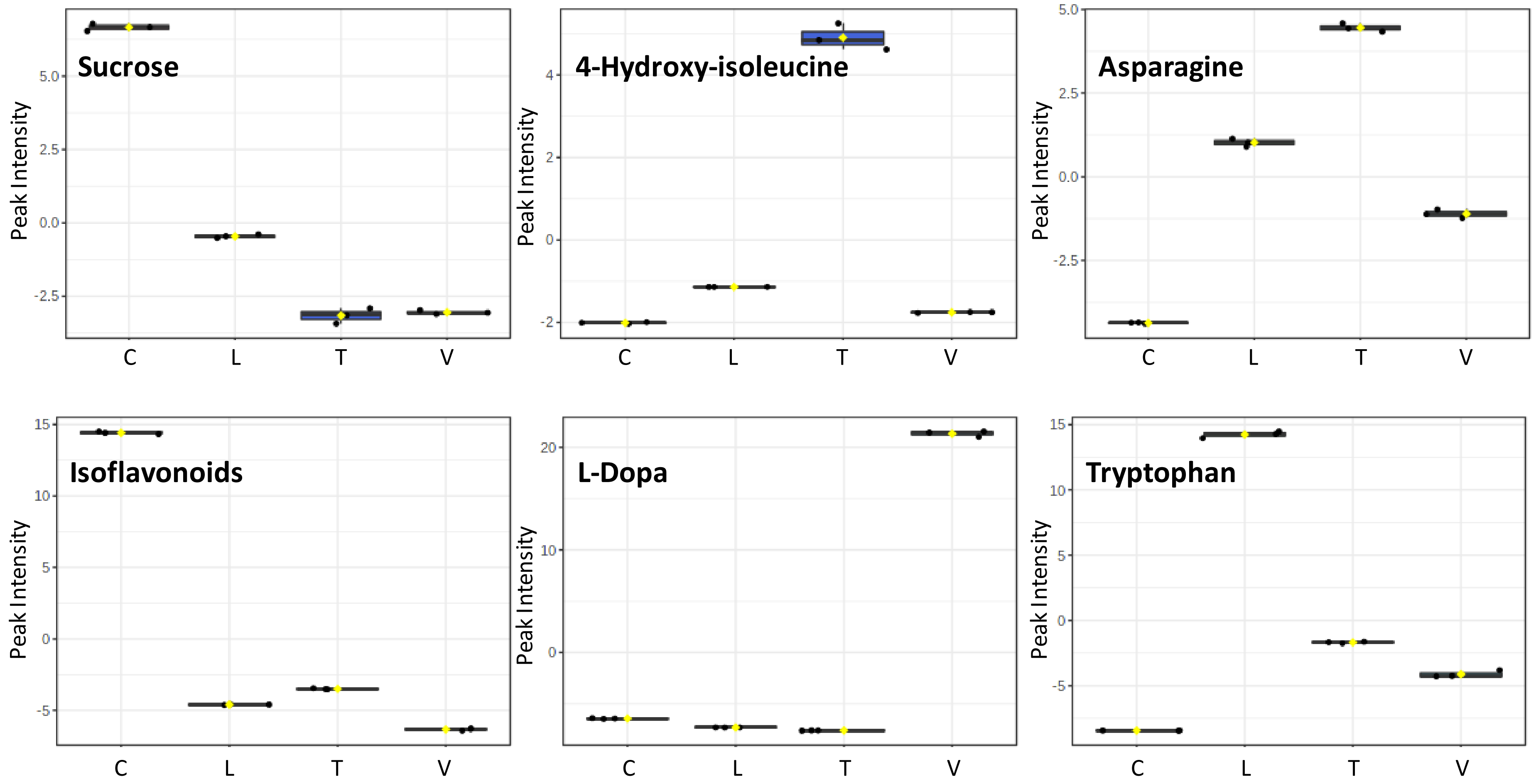

Figure. S14. Box plots showing peak intensities of sucrose, asparagine, 4-hydroxy-isoleucine, isoflavonoids, L-dopa and tryptophan in spouts (average of 3 biological replicates). These metabolites were identified by NMR and are responsible for the differentiation in PCA (line = mean; box = standard error; whisker = standard deviation). C, *Cicer*; L, *Lens*; T, *Trigonella*; V, *Vicia.*

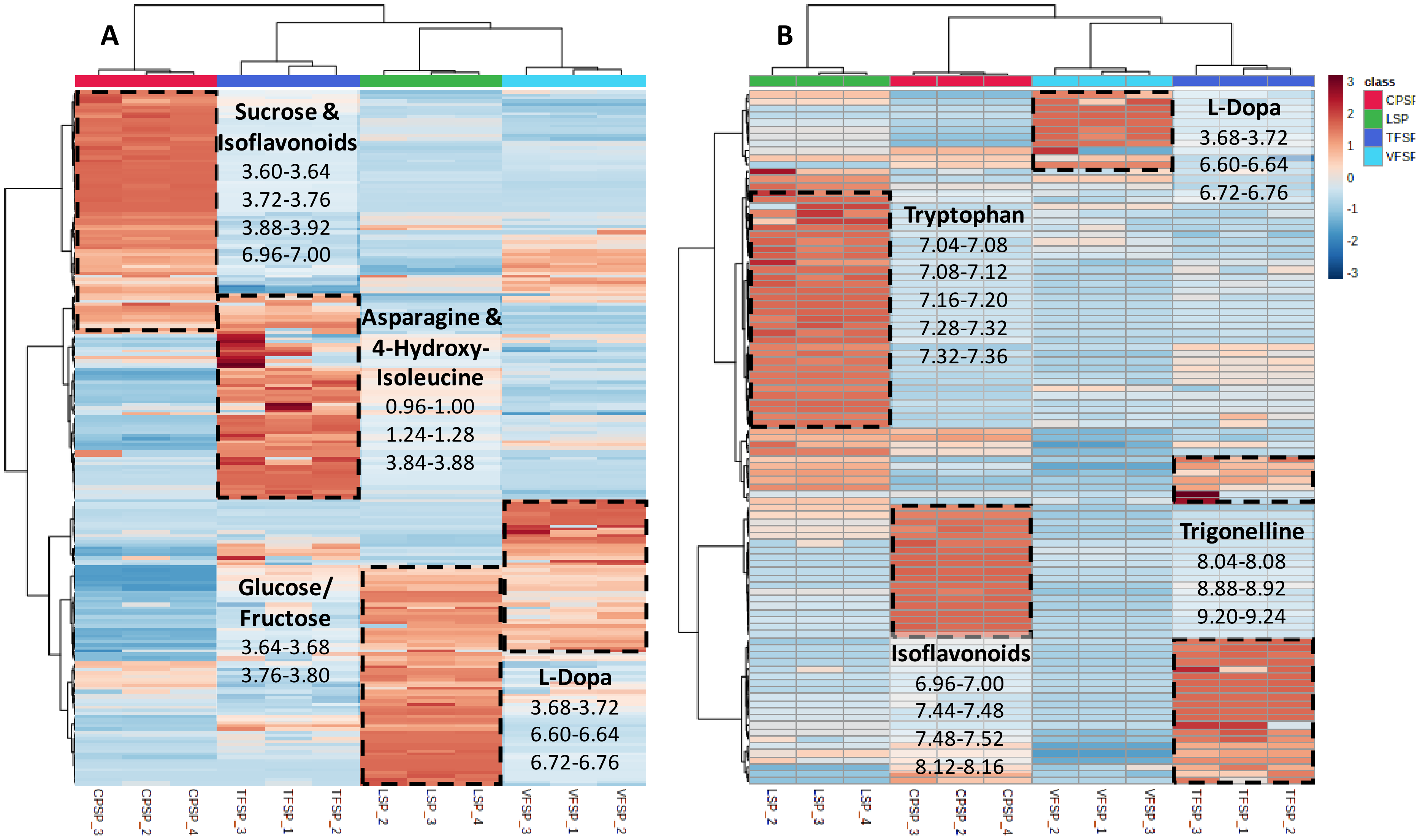

Figure S15. Heatmap plots of sprout samples based on group average cluster analyses of NMR biochemical profiles for **A.** full (δ 11.0-0.0 ppm) and **B.** aromatic (δ 11.0-5.0 ppm) regions, (n = 3). The color keys and histograms show the distribution of metabolites across sprouts.

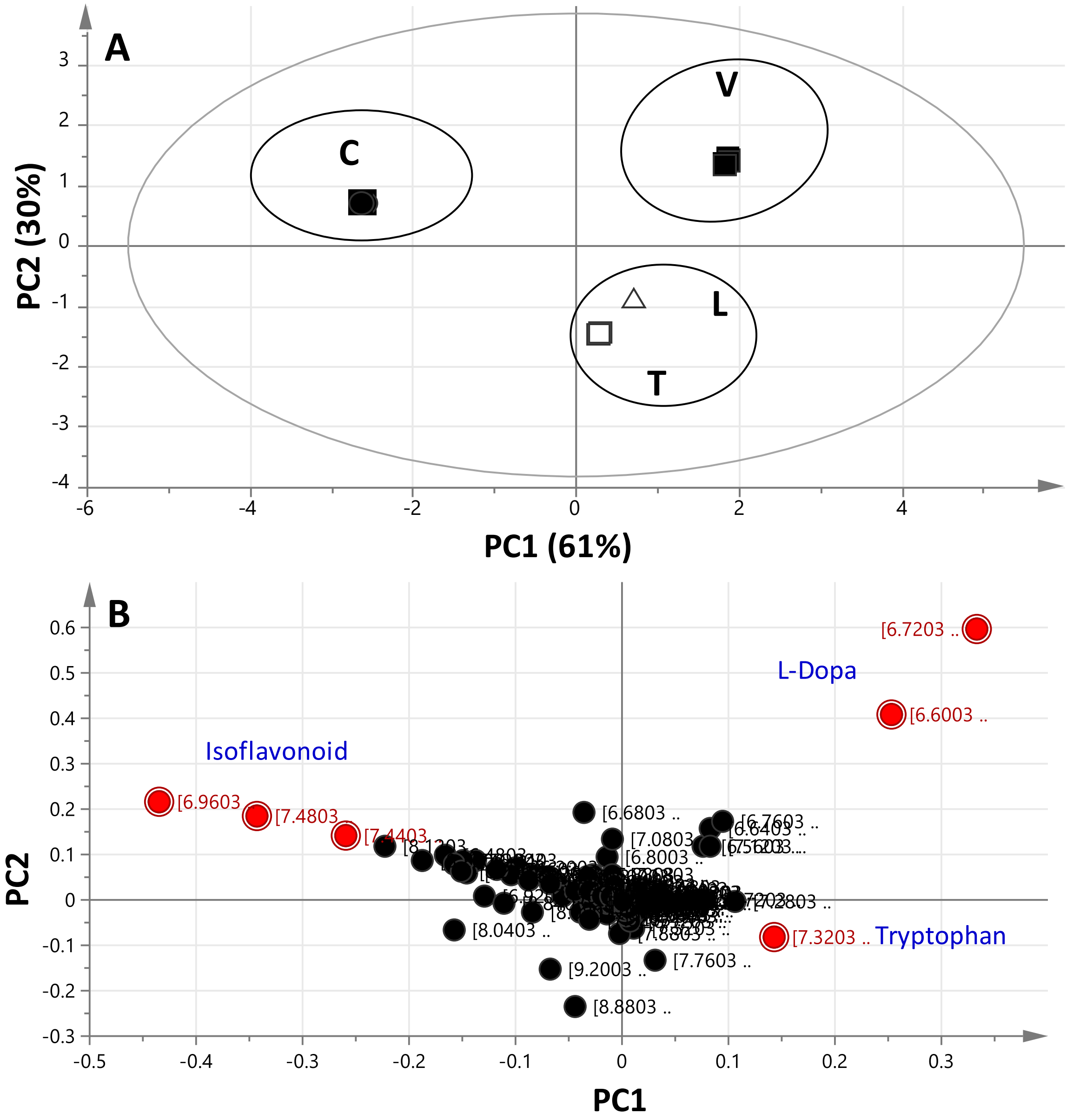

Figure S16. ^{1}H-NMR (δ 11.0-5.0 ppm) peak-based principal component analysis of the four legume sprout cultivars from *Cicer* (**C**), *Lens* (**L**), *Trigonella* (**T**), and *Vicia* (**V**) (n=3). The clusters are located at distinct positions in two-dimensional space described by two vectors of principal component PC1 (0.61) and PC2 (0.30). (**A**) Score plot of PC1 vs. PC2 scores. (**B**) Loading plot for PC1 & PC2 contributing ^{1}H-NMR signals and their assignments, with selected metabolites denoted by their chemical shifts (ppm). It should be noted that ellipses do not denote statistical significance but are rather added for better visibility of clusters discussed. For sample codes, refer to **Table 1**.

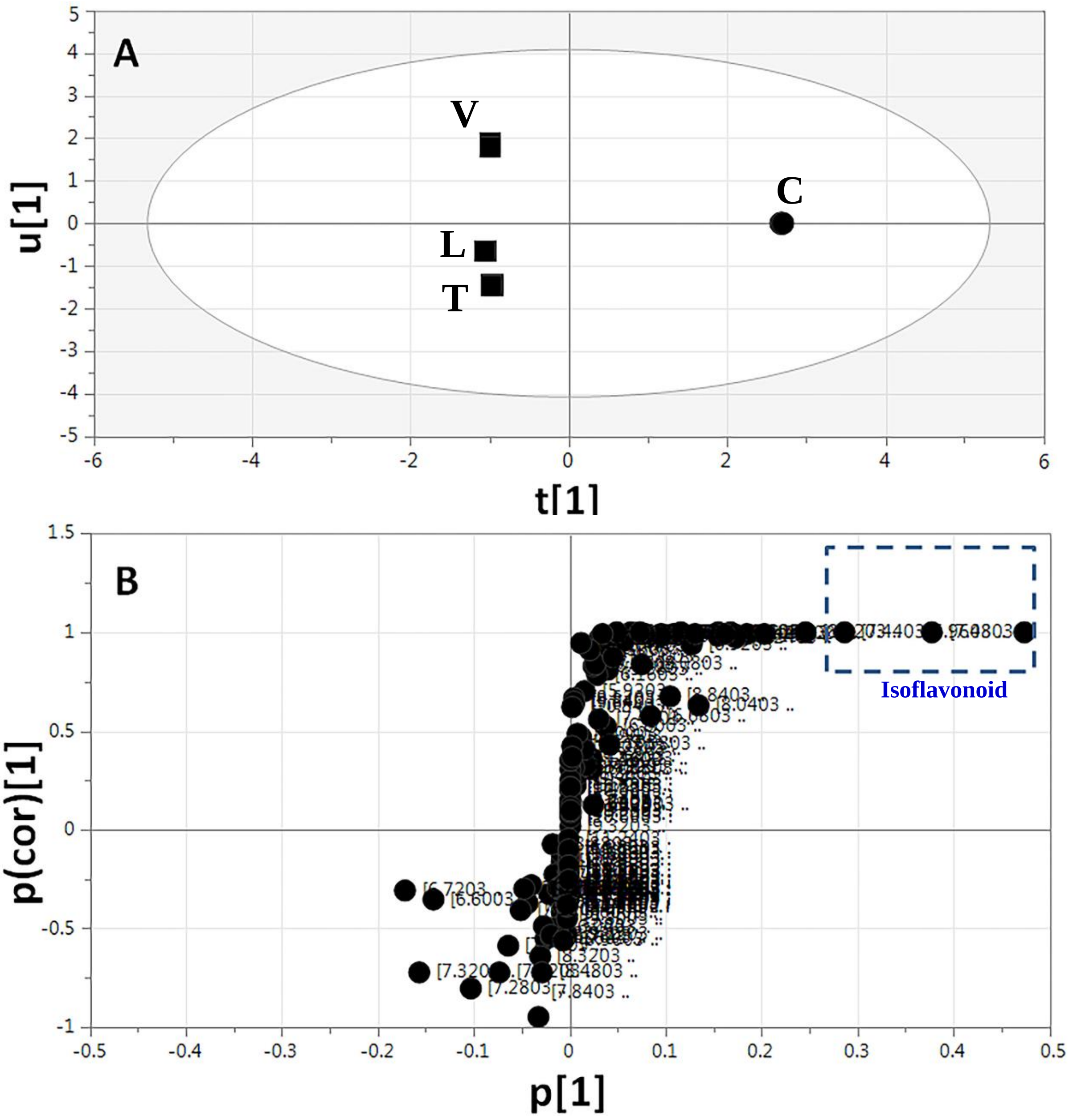

Figure S17. Orthogonal projection to latent structures-discriminant analysis (OPLS-DA) based on ^{1}H-NMR aromatic region (δ 11.0-5.0 ppm) of *Cicer* sprouts (●) modelled against the remaining legume sprouts (■) (n=3). (A) OPLS-DA score plot (B) loading plot derived from samples modelled against each other. The loading S-plot shows the covariance p[1] against the correlation p(cor)[1] of the variables of the discriminating component of the OPLS-DA model. Peak numbering follows those listed in Table **1** for metabolite identification using 1D-and 2D-NMR.

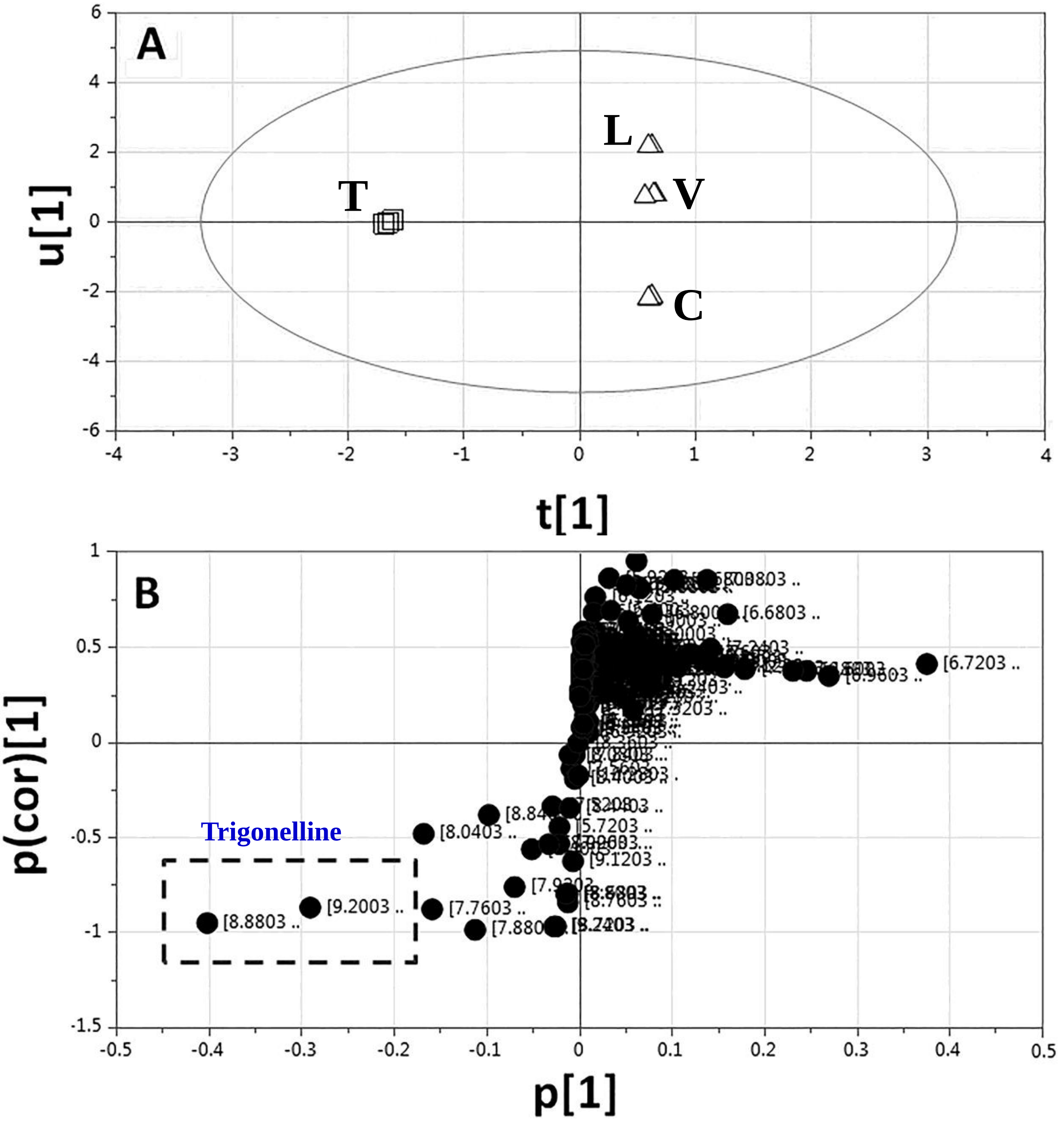

Figure S18. Orthogonal projection to latent structures-discriminant analysis (OPLS-DA) based on ^{1}H-NMR aromatic region (*δ* 11.0-5.0 ppm) of *Trigonella* sprouts (□) modelled against the remaining legume sprouts (Δ) (n=3). (A) OPLS-DA score plot (B) loading plot derived from samples modelled against each other. The loading S-plot shows the covariance p[1] against the correlation p(cor)[1] of the variables of the discriminating component of the OPLS-DA model. Peak numbering follows those listed in Table **1** for metabolite identification using 1D-and 2D-NMR.

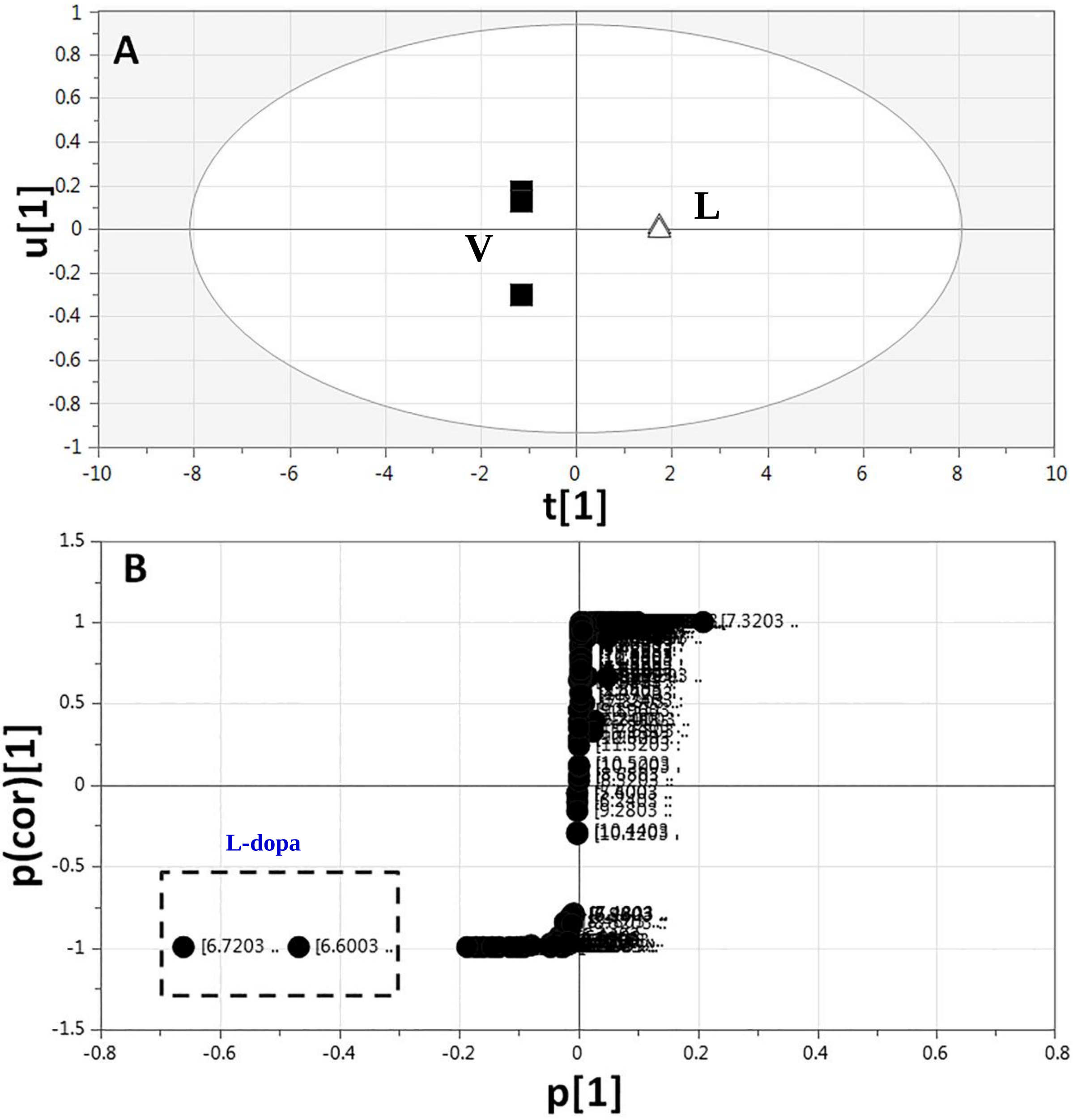

Figure S19. Orthogonal projection to latent structures-discriminant analysis (OPLS-DA) based on ^{1}H-NMR aromatic region (δ 11.0-5.0 ppm) of *Vicia* sprouts (■) modelled against *Lens* sprouts (Δ) (n=3). (A) OPLS-DA score plot (B) loading plot derived from samples modelled against each other. The loading S-plot shows the covariance p[1] against the correlation p(cor)[1] of the variables of the discriminating component of the OPLS-DA model. Peak numbering follows those listed in Table **1** for metabolite identification using 1D-and 2D-NMR.

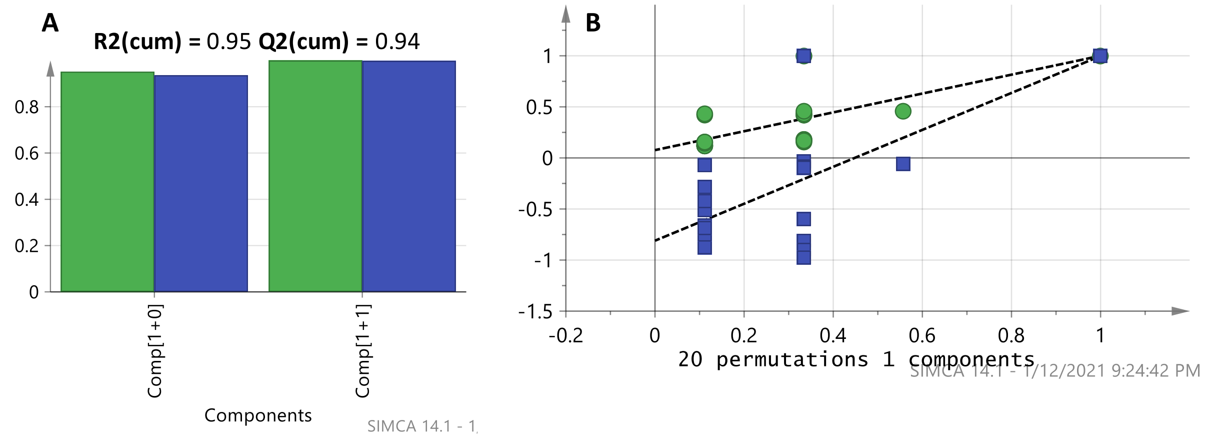

C

M1(Untitled)	SS	DF	MS	F	p	SD
Total corr.	11	11	1			1
Regression	10.9794	4	2.74484	931.7	0.00000000128202	1.65676
Residual	0.0206224	7	0.00294606			0.0542776

Figure S20. OPLS-DA model validation for modelling *Cicer* sprouts against other sprouts based on ^{1}H-NMR full region (δ 11.0-0.0 ppm) **A.** the diagnostic metrics R2 and Q2 **B.** permutation testing. n=20, and **C.** CV-ANOVA to assess for model statistical significance.

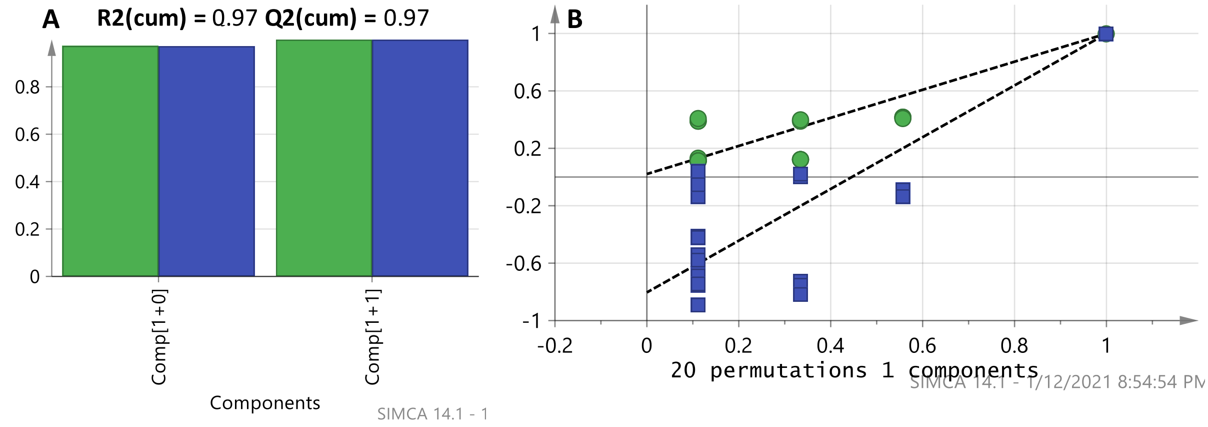

C

M4(Untitled)	SS	DF	MS	F	p	SD
Total corr.	11	11	1			1
Regression	10.9837	4	2.74593	1181.1	0.000000000559881	1.65709
Residual	0.0162743	7	0.0023249			0.0482172

Figure S21. OPLS-DA model validation for modelling *Cicer* sprouts against other sprouts based on ^{1}H-NMR aromatic region (δ 11.0-5.0 ppm) **A.** the diagnostic metrics R2 and Q2 **B.** permutation testing. n=20, and **C.** CV-ANOVA to assess for model statistical significance.

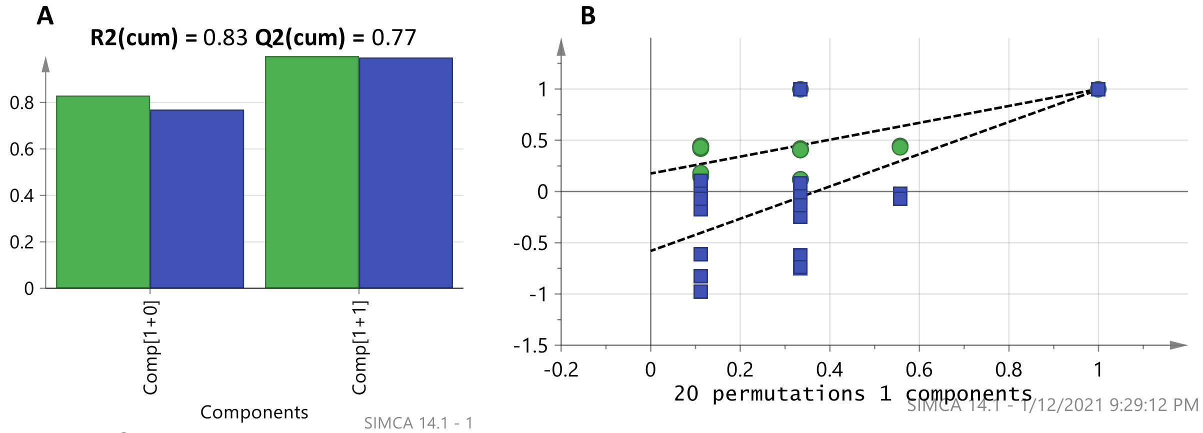

C

M3(Untitled)	SS	DF	MS	F	p	SD
Total corr.	11	11	1			1
Regression	10.9317	4	2.73293	280.117	0.0000000844481	1.65316
Residual	0.0682947	7	0.00975638			0.0987744

Figure S22. OPLS-DA model validation for modelling *Trigonella* sprouts against other sprouts based on ^{1}H-NMR full region (δ 11.0-0.0 ppm) **A.** the diagnostic metrics R2 and Q2 **B.** permutation testing. n=20, and **C.** CV-ANOVA to assess for model statistical significance.

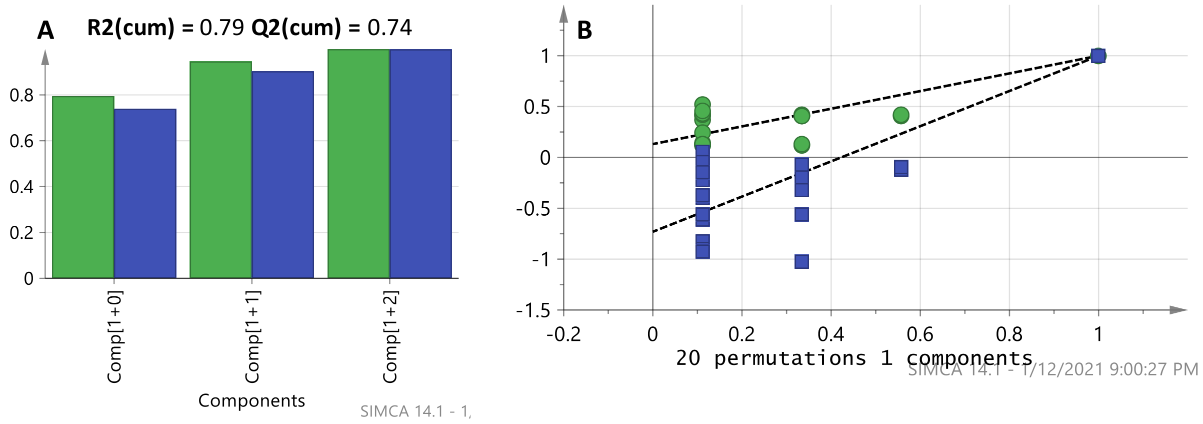

C

M4(Untitled)	SS	DF	MS	F	p	SD
Total corr.	11	11	1			1
Regression	10.9855	5	2.1971	910.442	0.000000014945	1.48226
Residual	0.0144794	6	0.00241323			0.0491246

Figure S23. OPLS-DA model validation for modelling *Trigonella* sprouts against other sprouts based on ^{1}H-NMR aromatic region (δ 11.0-5.0 ppm) **A.** the diagnostic metrics R2 and Q2 **B.** permutation testing. n=20, and **C.** CV-ANOVA to assess for model statistical significance.

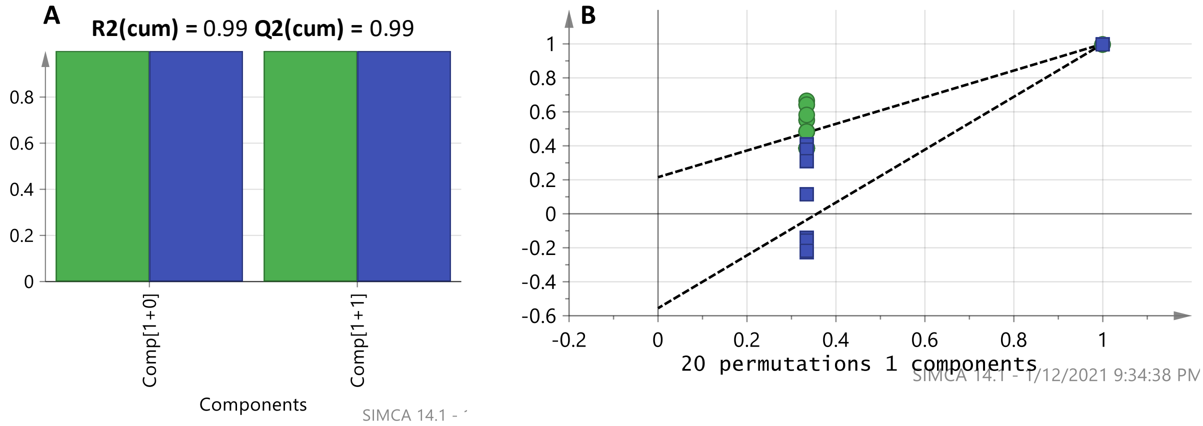

C

M4(Untitled)	SS	DF	MS	F	p	SD
**Total corr.**	5	5	1			1
**Regression**	4.9992	3	1.6664	4180.45	0.000239161	1.29089
**Residual**	0.000797235	2	0.000398618			0.0199654

Figure S24. OPLS-DA model validation for modelling *Vicia* sprouts against *Lens* sprouts based on ^{1}H-NMR full region (δ 11.0-0.0 ppm) **A.** the diagnostic metrics R2 and Q2 **B.** permutation testing. n=20, and **C.** CV-ANOVA to assess for model statistical significance.

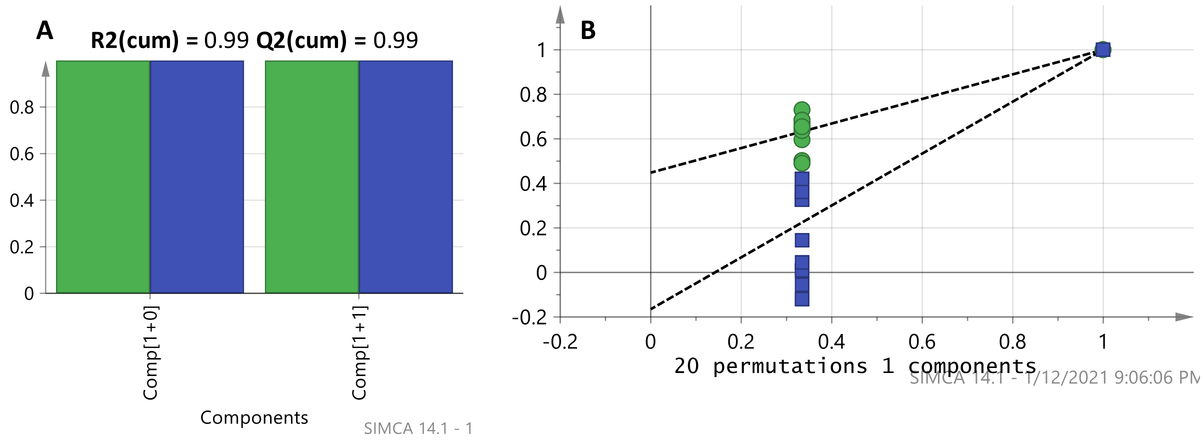

C

M4(Untitled)	SS	DF	MS	F	p	SD
**Total corr.**	5	5	1			1
**Regression**	4.99994	3	1.66665	56201.7	0.0000177928	1.29099
**Residual**	5.93095e-005	2	2.96548e-005			0.0054456<br>2

Figure S25. OPLS-DA model validation for modelling *Vicia* sprouts against *Lens* sprouts based on ^{1}H-NMR aromatic region (δ 11.0-5.0 ppm) **A.** the diagnostic metrics R2 and Q2 **B.** permutation testing. n=20, and **C.** CV-ANOVA to assess for model statistical significance.
